# A qualitative exploration of Irish nursing students' experiences of caring for the dying patient

**DOI:** 10.1002/nop2.1810

**Published:** 2023-06-05

**Authors:** Philip Hardie, Catherine McCabe, Fiona Timmins, David R. Thompson

**Affiliations:** ^1^ Hibernia College Merrion Rd Dublin Ireland; ^2^ School of Nursing and Midwifery, Trinity College Dublin Ireland; ^3^ School of Nursing, Midwifery & Health Systems University College Dublin Dublin Ireland; ^4^ School of Nursing and Midwifery Queens University Belfast Belfast UK

**Keywords:** death, dying, education, nursing student

## Abstract

**Aim:**

To explore Irish nursing students' experiences of caring for dying patients and their families to understand these experiences and determine whether or not students felt prepared for this role.

**Design:**

This study used a qualitative descriptive research design.

**Methods:**

One to one semi‐structured interviews were used to collect data, implementing open‐ended questions to explore seven student nurses' experiences.

**Results:**

Five main themes emerged: Student's first experiences, emotional experience of caring, educational preparation, challenging aspects of caring for dying patients and their families and need for support in practice. Students' first experience of caring for a dying patient and their family was a confronting event for students, both personally and professionally. Nursing students require adequate and timely education on end of life care and a practical and supportive clinical learning environment to effectively support and prepare students for caring for a dying patient and their family.

## 1 INTRODUCTION

Nurses play a key and substantial role in delivering end‐of‐life care (McCall, 2018). Therefore, student nurses will likely encounter dying patients and in some cases death at an early stage of their nursing education on practice placements in the hospital. However, providing care for dying patients and their families is one of the most challenging aspects of nursing (Hançer Tok & Cerit, 2021), particularly emotionally for nurses in practice (Zheng et al., 2018). Several studies have identified that when nurses do not receive an adequate end‐of‐life education, feelings such as death anxiety and a negative approach to care have been reported (Wang, 2019). End‐of‐life care can be complex in nature, focussing on meeting the patient's physical and emotional, social and spiritual needs (NICE, 2019; WHO, 2020). Although nursing students are typically supported by their preceptors, many still find this experience distressing (Garrino et al., 2017). Witnessing pain and suffering and the risk of sudden death or unexpected adverse events are key issues of concern for students during this time (Henoch et al., 2017). Students can also lack confidence in caring for the dying and their families (Wang, 2019). Gillan et al. (2021) recently explored nursing students' experiences of in‐hospital death in Australia and found the experiences varied: students supported by nursing staff who were able to learn from them and model their behaviours had good experiences. However, there were also negative experiences related to ritualistic and undignified care, particularly for the physical aspect of death, such as the coldness of the body and post‐mortem examination (Gillan et al., 2021). To enhance the preparedness of undergraduate nursing students to participate in the safe and effective provision of end of life on their clinical placements, educators continue to strive to advance students’ education. Recent pedagogical practices to teach nursing students end of life care include simulation (Smith et al., 2018), films and problem‐based learning (Hökkä et al., 2022). However, despite significant advancements in the quality of the education and training of nurses in recent decades, gaps exist concerning preparation for this crucial area of practice.

## 2 BACKGROUND

Personal attitudes to death and dying contribute heavily to nurses' behaviours around dying, including personal attitudes to cultural rituals, religious factors and spiritual superstitions. Examples include concern about being ‘challenged by a deceased person's soul or ghost’ when caring for the dead (Shih et al., 2006), therefore the opening of the window in the room to ‘let the spirit go free’ and speaking to the patient who has died as if they are still living (Roxby, 2012). In contrast, Kudubes et al. (2021) recently found that nurses with negative attitudes towards death and cultural rituals were less likely to provide spiritual care to dying patients.

Social norms related to death and dying vary across cultures and over time. For example, death customs in the Republic of Ireland arise from Christian and Pagan beliefs (Ridge, 2009). Christianity informed old customs such as the requirement for *three* women to ‘layout the dead and light a candle so that the deceased was never in darkness’ (O'Suilleabhain, 1997). Traditional Irish ‘wakes' often lasted for days involving alcohol, dining, joking, weeping and insulting the deceased (Muir, 1997). Religious practices increasingly replaced such events (Taylor, 1989). However, with increasing secularization, the nature and importance of death rituals have declined (Hyde et al., 2004; Walter et al., 1995), although modest ‘wakes' have retained some popularity. At the same time, although most people express a preference to die at home, death is increasingly an in‐hospital experience (Westwood & Brown, 2019). Additionally, a natural death is not always possible in modern society, as medical interventions to prolong life are often a necessary but sometimes controversial component of modern health care (Gwande, 2014; Heyland et al., 2015). As such, the cultural manner of death is changing and becoming an increasingly clinical experience, fraught with ethical dilemmas, sometimes devoid of spiritual or existential support and not necessarily the expected dignified death (Gwande, 2014). Witnessing death is also becoming a relatively novel experience. For people who were once accustomed to the visceral nature of death and suffering, changing customs, improved treatment and reduced mortality rates mean that many nursing students experience death and suffering in the clinical sites for the first time.

Factors such as age, previous exposure to death and education have all influenced nursing students' attitudes towards the care of dying patients (Berndtsson et al., 2019). Several studies have investigated student nurses' experiences of caring for dying patients and their families around the world, including Australia (Ranse et al., 2018), Sweden (Ek et al., 2014; Österlind et al., 2016; Strang et al., 2014), the UK (Grubb & Arthur, 2016), Italy (Garrino et al., 2017) and Nigeria (Faronbi et al., 2021) finding death and caring for dying patients and their families can often be a difficult and emotional experience for students. However, research has not yet investigated Irish student nurses' experiences caring for dying patients and their families. Therefore, this study sets out to examine the following questions:

- What are Irish student nurses' experiences caring for dying patients and their families in the clinical setting?
- Do Irish student nurses feel adequately prepared to carry out this role?

## 3 METHODS

### 3.1 Design and setting

This study used a qualitative descriptive research design undertaken in a large university in Ireland, which offers undergraduate degree programmes across nursing and midwifery.

### 3.2 Ethics statement

This study was approved by the Local Research Ethics Committee. A gatekeeper emailed the students on behalf of the authors, notifying students about the scope and aims of the study and their involvement if they decided to partake in the study. Over 2 weeks after receipt of the email, seven students volunteered to partake in the study via return email, and written consent was obtained. The researcher completing the interviews was unknown to the students. Full confidentiality, privacy and the right to withdraw were permitted.

### 3.3 Participants and setting

This study adopted a purposive sampling approach recruiting students from the penultimate year (4th year) of their programme to ensure adequate exposure to the phenomena. An eligibility criterion included students who had cared for at least one patient: (a) who had died, (b) diagnosed with a terminal illness throughout the time the student was caring for them and (c) cared for but who died from a respiratory or cardiac arrest. An invitation to participate was emailed to all 4th year (*n* = 80), nine responded, and seven students met the criteria and were interviewed. Data saturation was achieved from the seven participants. Therefore, the researchers did not seek to recruit any more participants. All were female participates, White Irish and between the age of 20 and 35 yrs.

### 3.4 Data collection

One‐to‐one recorded semistructured interviews lasting 30–45 min were used to collect data by one research team member. One‐to‐one interviews were selected to gather data due to the sensitive nature of the topic being discussed and to ensure the confidentiality of the participants involved in the study and the patients and families described throughout their experiences (DeJonckheere & Vaughn, 2019). An interview guide was developed utilizing Kallio et al. (2016) framework for a qualitative semistructured interview guide, including pilot testing the guide and presenting the guide to all students prior to the interview. The interview guide supported this process by incorporating open‐ended questions, for example Can you tell me about your experience caring for a dying patient and their family during your clinical placement? Probing questions to seek further detail or better understand the students' experience was used, e.g. ‘Could you tell me more about that’ or ‘Can you please explain what you mean’. However, the progression of the interview depended on the participant's answers, thus allowing participants to have control and freedom in the interview to express their experiences and opinions. Due to the topic's sensitive nature, students were advised that they could stop the interview at any point if recalling their experiences was emotionally distressing and that student counselling services were available if required. Following the interviews, the author summarized key theoretical ideas that occurred throughout the interview, such as death anxiety and experiential learning. Finally, the researcher noted their response to the student's experiences and comments, acknowledging that they had been exposed to challenging situations but that all students conducted themselves professionally and empathetically despite their fears and anxieties.

### 3.5 Data analysis

Interviews were transcribed verbatim, and immersion of the data was achieved through repeated listening and reading of the audio transcripts. To identify, analyse, organize and describe key themes, commonalities and differentiation among the individual experiences of the participants, an inductive thematic analysis using Braun and Clarke (2006, 2021) six‐phase framework was applied: Step 1: Familiarization, Step 2: Coding, Step 3: Generating initial themes, Step 4: Developing and reviewing themes, Step 5: Refining, defining and naming themes, Step 6: Writing up. The process of reflexive thematic analysis is not strictly linear, resulting in a recursive process. Each transcript was read simultaneously while listing to the audio recordings, enabling the authors to note the participants' speech, tone of voice and word articulations. These data were manually colour‐coded to identify critical points and associated feelings. Each critical point was then organized into themes and used to describe the students' experiences. The themes were again reviewed, and the data were associated with each theme to ensure the data did support the themes identified and whether the themes worked in the context of the entire data set.

### 3.6 Rigour

To ensure credibility and reduce any possibility of bias in the researcher's interpretation of the data, transcripts were returned to and approved by the participants to ensure that they recognized these experiences as their own (Polit & Tatano Beck, 2014). All students were satisfied with the interpretation of the data. Credibility was also achieved by selecting the most suitable meaning unit and developing categories and themes that are extracted as relevant to the main findings (Bengtsson, 2016). Dependability was assured by having a single researcher perform all the interviews in a consistent fashion (Graneheim & Lundman, 2004). Transferability was achieved by synonymizing the ultimate findings with the literature on the topic.

## 4 FINDINGS

Five main themes emerged from the data: students' first experience, the emotional experience of caring, lack of educational preparation, challenges with communication skills and the need for support in practice.

### 4.1 Student's first experiences of caring for a dying patient and their family

Students vividly described their first experiences caring for a dying patient and their family on clinical placement in diverse hospital settings from the care of the elderly ward, renal ward and Accident & Emergency Department (A&E). Students described varied experiences of death ranging from cardiac arrest to terminal illness.

Well, the first that I was involved was in A&E, and they got the call that there was a cardiac arrest coming in…….They said he could be dead on arrival. The patient came in anyway; they started CPR. The staff pretty much got the student nurses in there to get us to experience CPR because they didn't think that we would revive him. Nothing was successful #student 2.

I was looking after an old lady who was dying in a private room, she didn't have cancer or anything, she had dementia, she was deteriorating at home, and they brought her in, and she was put on an end of life pathway and was on a morphine pump, and there was no specific reason why she was dying, she was just old #student 7.

One of the students expressed that she felt utterly unprepared and more of a bystander in the situation:

There was no difference between me, and if I was on the side of the street and the paramedics were doing it, I was just like a by‐passer. I just felt like I was in the way the whole time. I was purely just observing what was going on. Looking back at it, I don't even know if I fully took in what was going on #student 1.

### 4.2 The emotional experience of caring

The students talked about a broad range of emotions they experienced while providing care to dying patients and their families, including dignity, empathy and compassion. However, many students stated that caring for dying people evoked unpreparedness and uncertainty, resulting n physical manifestations of fear, panic and sadness.

It was kind of sad because I'd never looked after anyone who was dying before, …., I found it really upsetting #student 7.

It was quite upsetting it was the first real hands one death that I dealt with…I just found it was upsetting in general, I think at first you don't really know how to deal with it, you don't actually know until you have done it’ #student 3.

I was very sad, and I was panicking …. I just felt really out of my depth #student 5

Sometimes it was not the event of death that caused sadness but rather the family's reaction to it:

I kind of had a lump in my throat that I was trying to hold back because they're not your family, but I suppose you empathize with the people you see because they're so upset because it's their loved one, and I suppose you can picture yourself being in their shoes or you can relate it to an experience you have had before. I suppose it's more empathy for them because it's such a hard situation to deal with. I was upset myself; I was sad myself #student 4

Students sometimes reported feeling a dissonance between the dignified and orderly care that they would have expected and the realities of practice at the time of death:

[They weren't] very nice to the family, and I found that quite upsetting because I felt it could have been dealt with a lot better #student 3.

He was kind of treated like a piece of meat [in the emergency resuscitation room], and all the cannulas were put in; they weren't really respectful of the body. I felt so awkward there. It wasn't nice. . I felt bad because I didn't know if I should stop or not (CPR). I find it hard to think of him as a person because that's just kind of horrible. I find it hard to think that because the rest of the team were just there doing the skills. #student 2.

In some cases, students reported a professional detachment concerning dying using distance language when describing their experiences. For example, ‘she was old age, and ‘to get us to experience CPR’, ‘I wasn't even very close to it’. Another student reports being a bystander and not taking it in, while another stated ‘I think you learn not to be so attached’.

Some students spoke about further experiences caring for dying patients and their families. Within this context, it was clear that although their experiences were difficult and upsetting for some students, positive experiential learning opportunities arose, and they began to feel more confident in their roles. They developed coping mechanisms and a greater competency in caring for a dying patient and their family:

I think you learn not to be as attached. There will always be certain people who will catch a bit of your heart that you can't help but feel for them and be upset when they pass away. However, there are other people, and I think most of the time you have to have the professional distance while doing as much as you can for them. I think that is something you learn as you go along; you learn to detach yourself #student 4.

You learn more each time, ways of coping with it, and it will still get to you, but you just learn to rely on the other staff members and your friends, and just chat about it. Overall, every aspect of communication, naturally dealing with the body, even things like the paperwork where someone is going to need a post‐mortem done, the paperwork for that, the family need to go to the mortuary to see the patient, breaking bad news #student 2.

### 4.3 Educational preparation

In terms of preparation for supporting families and patients at the end of life, the students referred to their educational preparation received within the university. Students discussed receiving lectures on ‘the last rights’, ‘different cultures and dying’, ‘palliative talks from the chaplain’. However, many believed the timing was inadequate to prepare them for practice as it often came too late.

I think my first dying patient was in 1st year, but I remember getting all the palliative talks‐ getting the chaplain etc. I don't think we got that till 4th year #student 4.

It was all after I personally had done it, but that's just the way our placements work out…… Personally, I would have preferred to have them before….. it's just so you could be a bit prepared. I then would have had the full background….you have an idea what's going on, and maybe I wouldn't have felt like I was just watching. I literally felt like I was watching a football match. I had no idea what was going on #student 1.

The education and preparation for supporting death and dying appeared limited, and students believed that the didactic lecture‐type approach to learning they had received was not the most optimal to provide the necessary skills and competencies required in real‐world situations articulating the desire for a more active form of learning, such as role plays:

Yeah, maybe some role play, not just a lecture on communication that's not really that helpful when you're in the situation to be thinking about what did the lecturer say #student 3.

Definitely practical. I think sitting down in a lecture, going through slides is not working. I think that scenarios should be brought in, and a lot of other things should be brought in practically #student 5.

### 4.4 Challenging aspects of caring for dying patients and their families

Students described various elements they found difficult for caring for dying patients and their families, including providing holistic care, laying out the body and interacting with the family and other patients on the ward during that time.

One student described how she has only had one experience to date but also commented on how as a student, she tended to avoid the situation:

To date, I've only had one, that's from 1st to 4th year, and I wasn't even very close to it. As student nurses, we kind of ran away with it more so than faced it #student 6.

Some of the students described challenging ethical considerations in the end‐of‐life care experiences they were involved in, including resuscitation, terminal sedation and pain management in palliative care. One student told the challenges from an ethical perspective where a compos mentis patient requested withdrawal of care and not for resuscitation. But the family disagreed with the patient's decision resulting in high stress for the patient and anger for the family.

The family can be difficult if angry, they're not angry at you but at the situation. They [the family] were asking me questions like: Have you done this ? Why can't you do more? I felt bad for the patient and the family because I could see it was causing her [the patient] a lot of stress and the family just didn't want to lose their mum, but you have to respect the patients wishes #student 7.

Most students described communication as the most challenging aspect of caring for dying patients and their families.

For example, one of the students described how she found it very difficult to communicate with the patient if they did not know their diagnosis. This limited the type of communication the student engaged in, and they kept it at a superficial level:

Answering his direct question, he seemed to be aware of it, but it's that boundary of had he been told? Is he aware of it? Have the doctors given it? It's not handed over in handover; If he was aware of his diagnosis, you could say, "you are, but you're doing fine is there anything I can do to make you feel more comfortable". I think it would have given you a better dialogue to deal with but, when [they don't know their diagnosis] – what are you to do then? #student 6.

Most students described challenges communicating with the family:

Well, they were asking me if he was going to die in the next few minutes? I was scared to say yes because I didn't know. I just didn't feel comfortable at all answering the question; I just was so anxious to get out of the room. I still think now, in 4th year, that I still would not be that confident in talking to a family on my own. I think I would be scared to say something, and then a staff nurse says, oh, you shouldn't have said that. I'd be afraid I'd say the wrong thing. I still wouldn't be confident #student 5.

Communicating with other patients on the ward after death was also difficult:

The other patients as well. Especially somewhere like the stroke unit – all the other patients were there had had a stroke, so a lot of them were like, "Oh God is that going to happen to me? #student 1.

### 4.5 The need for support in practice

Some students accounted for getting little support from their preceptors throughout the experience of caring for a dying patient and their family. They described how the preceptors were not supportive and unapproachable.

Some students described receiving limited support and guidance:

On that particular ward, I had an awful preceptor. [They were] unapproachable .. so .. I did not get support #student 6.

Nobody was explaining what was going on, he was just my patient, I was to deal with it #student 2.

The supportive approach used was not always optimal:

[They] did a group session with us afterwards because she heard that someone had died when she was doing her rounds, and then she took us for reflection… But I'm not really comfortable speaking out when there are big numbers of people, so I probably would find it better if it was a one‐to‐one session #student 2.

Alternatively, other students gave various accounts of receiving great support from senior nursing staff and colleagues.

They were really, really good; I know that when I was in 1st year to the point that they asked if I wanted to go home. They said I'd seen a lot that day. Definitely, [staff] were really good as well in so far as providing numbers and contacts for the college counselling system, so that was really, really good #student 1.

Equally, informal networks of colleagues proved to be a valuable source of support:

I did go and talk to my friends in the staff room after and said how I felt after, and I suppose that was my counselling…it was good to share my experiences with other people, so that was my kind of therapy #student 1.

## 5 DISCUSSION

The student narratives from this study identify the complex experience that caring for dying patients and their families can be for student nurses. Irish student nurses described strong emotional feelings and a lack of clinical preparedness and competence in line with previous literature. Like Gillan et al., (2021:3) study, student participants remembered their first death ‘like it was yesterday’ students' experience of death in this study was easily and vividly recalled. Similar to previous studies in Australia, the UK, Italy and Sweden, Irish students described experiencing death and dying in a variety of clinical settings and at various stages of their undergraduate programme (Cooper & Barnett, 2005; Ek et al., 2014; Grubb & Arthur, 2016; Österlind et al., 2016; Strang et al., 2014) suggesting care of a dying patient and their family is a core nursing responsibility and skill and is required for all clinical settings from an early stage of a nurse's career.

The initial clinical experience and contact with death and dying are well‐understood as significant sources of stress for students, especially during early clinical exposure (Yoong et al., 2023). Findings from this study suggest that student nurses developed more positive attitudes towards the care of dying patients and their families at later stages of their undergraduate programme, following clinical exposure and experience. This is consistent with findings from other studies (Grubb & Arthur, 2016; Henoch et al., 2017; Sharour et al., 2017; Zulfatual et al., 2018). Further research incorporating a longitudinal study investigating student attitudes to death and dying would be beneficial to understand how their attitudes develop over time.

Poor communication skills can affect patients and their families during end‐of‐life care (Caswell et al., 2015). ‘Good communication of a dying person's prognosis improves patients' end of life care and the bereavement experience of those important to them. It can help to ensure that the dying person's expressed wishes are considered and to avoid misunderstandings and unnecessary distress’ (NICE, 2015:8). Nevertheless, a common theme emerging from most students in this study was their reported difficulties and anxiety communicating with dying patients and their families. The literature supports this finding with students often feeling unprepared and unsure of what to say in such circumstances (Parry, 2011), and stress can arise due to a lack of confidence in their communication abilities (Smith‐Stoner et al., 2011).

Nursing students frequently report that they are at the forefront of ‘serious illness’ (Le et al., 2021), frequently receiving questions such as, ‘*nurse, am I dying*?*’*. They often feel ill‐equipped for such conversations and are shocked to find themselves in serious dialogue with patients struggling with responsive reactions. Nursing students may find themselves in such situations as nursing staff often lack the time (Le et al., 2021). However, a lack of education, training and support for hospital leadership can make such conversations challenging even for qualified staff (Le et al., 2021). Wu et al.'s (2019) systematic review revealed that in some incidences, although students had learned and practised communication skills in the university setting, they felt ill‐equipped to speak to patients about a poor prognosis or length of life left. However, it has also been found that nursing students who are more advanced in their programme are more comfortable with communication (Grubb & Arthur, 2016), indicating that skills development and building with experience are something that students in our study certainly experienced. Further research is required to investigate the direct impact communication skills workshops dedicated to end‐of‐life care have on a student's ability to communicate with a dying patient and their family.

Interestingly, this study found that most students received education about death and dying after they had already experienced death. Moreover, their education often focussed on the physical aspects of caring for the dying patient, with little or no consideration of the psychosocial aspects of care or students' self‐care. Other studies reveal that nursing students' knowledge about end‐of‐life care is poor; particular areas of weakness are knowledge about pain/symptom management and psychosocial/spiritual care (Dimoula et al., 2019), suggesting that a greater focus is needed on communication, misconceptions and biases and providing comfort (Dimoula et al., 2019). The students in this study felt that the education and preparation to support caring during death and dying appeared limited and believed that the didactic lecture‐type approach to learning they had received was not the most optimal to provide the necessary skills and competencies required in real‐world situations. Gillan et al. (2014) found similar results, highlighting that didactic modes of delivery fail to provide students with opportunities to examine their reactions to death and dying. Equally, a more recent study in Sweden found few undergraduate nursing programmes include a specific course about end of life care in their curricula (Hagelin et al., 2022), suggesting a lack of priority on teaching end of life care as part of the undergraduate curricula internationally.

As previously discussed, caring for dying patients and their family is an emotional time for students; they must receive education and support to reduce moral distress. A recent systematic review revealed that educational interventions could positively affect nursing students' attitudes towards death and dying, although specifically more beneficial to understanding and supporting the dying process than death itself (Chua & Shorey, 2021). They point out that variances in personal beliefs, such as religious beliefs, can influence the reactions and effects of education (Chua & Shorey, 2021) and might outline how vital self‐awareness is in this area. Interestingly, Chua and Shorey (2021) and Zhou and Wei Zhnag (2020) also suggested that future interventions ought to include ‘spiritual components’ to ‘enhance the impact of educational interventions on attitude toward death’ (Chua & Shorey, 2021:6). It is not clear the exact nature of this relationship; however, personal beliefs might increase positivity towards death, for example, through a belief in an afterlife, or that supporting patients' spiritual needs provides solace and something to do at this time. Further research examining the impact of students' personal beliefs on end of life care is required.

Moreover, this study examined whether student nurses felt prepared to carry out end‐of‐life care on their practice placements in the hospital. The students in this study demonstrated emotional reactions and uncertainty around competency, despite receiving basic instructions in nursing care, including foundational communication skills workshops. Furthermore, end‐of‐life care skills taught by lectures failed to prepare students for the realities, especially the emotional aspects of caring at the end of life. Procedural skills only equip the students to an extent. The realities of seeing a person who has died, the coldness of the body and how the families' reactions affect the student are not transmitted in regular clinical teaching and could be emphasized within these newly emerging simulations. Indeed, students report them as ‘bridge‐building between theory and clinical practice’ (Honkavuo, 2021:3). Overall, their purpose is to ‘convey to participants a narrative experience or an interpretation of the meaning of the simulation, which simultaneously strengthens possible empathic and emotional experiences’ (Honkavuo, 2021:3). Nursing students must receive timely, responsive end‐of‐life care educational experiences that support ongoing reflective practice. Approaches to teaching for nursing students must aid an understanding of patients' suffering. Relational inquiry approaches can help develop a deeper understanding of patient suffering by ‘building a therapeutic and trustworthy relationship, active listening, focusing on the details, and engaging in broad and situations specific inquiries to understand the patient narrative of suffering’ (Younas, 2020:934). Coping with death is helped by setting boundaries, reflection, crying, death beliefs, life and work experience, and daily routines and activities (Zheng et al., 2018). Other supportive actions comprised talking and receiving education and debriefing. The literature supports these approaches (Zheng et al., 2018). Localized debriefing and reflection on death experiences might be a valuable adjunct to students' learning in the clinical area. Reflective practice is key to this learning and is supported in the literature (Chua & Shorey, 2021; Westwood & Brown, 2019).

Given the reality shock and emotions often experienced by these students, it is clear that future teaching needs to include preparation for the realities of practice. Education of nursing students needs to include ‘meaningful preparation’, including preparation for post‐mortem, ethical considerations and the possibility of ‘bad deaths’ (Gillan et al., 2021:97). Berndtsson et al. (2019) recommend subjects such as communication skills, death and dying and bereavement, and the essential practical nursing skills to function on the ward are essential prerequisites to entering practice for the first time. Participants in this study expressed a desire for more responsive and realistic teaching methods. Ethical simulation laboratories are essential for facilitating such learning in educational settings (Honkavuo, 2021). In addition to imparting the necessary clinical skills, these laboratories focus on the complexities and ethical dilemmas of practice and, thus are likely more sensitive to the needs of students when learning about end‐of‐life care. Jablonski et al. (2020) found that providing students with the opportunity to experience emotions evoked by the end‐of‐life care simulations in a safe setting positively influenced students' preparation for the end‐of‐life care in the clinical setting. Jablonski et al. (2020) also established that incorporating a ‘family member’ into the simulation and performing post‐mortem care contributed to the reality and effectiveness of the simulation.

Lippe and Becker (2015) describe the positive impact that running simulations on the withdrawal of care had on students' attitudes and perceived competence in caring for a dying patient. Similarly, Hançer Tok and Cerit (2021) identified that implementing creative drama workshops focussed on death and dying patients and reflection on artworks (Nicol & Pocock, 2020) positively impacted student learning and students' attitudes towards caring for dying patients indicating perhaps the need for more creative approaches to learning. The literature suggests innovative educational practices alongside supported clinical experience will offer students the best opportunity to feel comfortable and actively participate in the end‐of‐life care. Future research is required to examine the direct impact of simulation education and creative pedagogy on students' competency in providing patient and family centred end of life care.

### 5.1 Limitations

This study employed a purposive sample from one educational institution. A small sample size and an all‐female population restrict the ability to generalize the findings of this study to the broader student nurse population. However, this sampling approach was consistent with achieving the aim of this study to explore and gain insight into Irish nursing students' experiences of caring for dying patients and their families.

## 6 CONCLUSIONS

The findings from this study confirm that Irish student nurses' experiences of death and dying are similar to those previously reported internationally, with many students describing emotional and stressful experiences. However, a key implication for nursing education that emerged from this study was a clear need for realistic, responsive education on communication with dying patients and their families, exploring students' personal beliefs and cultural components of end‐of‐life care and ethical considerations of end‐of‐life care. Students themselves reported that the traditional didactic approach was unsuitable, and a more active approach to learning needs to be implemented in a timely fashion in advance of their practice placements. Yet, for experiential learning to be positive and supportive for students, guided reflection and debriefing sessions should be provided as this may prove effective in giving students confidence. This activity will enhance self‐awareness and the ability to recognize and help students manage their emotions. Finally, student mentors and educators in practice may also require adequate education to support the students and become effective role models in caring for dying patients and their families.

## AUTHOR CONTRIBUTIONS

All authors have made substantial contributions to all of the following. PH and CMC were involved in the conception and design of the study, or acquisition of data, or analysis and interpretation of data. PH, CMC, FT and DT were involved in drafting the article or revising it critically for important intellectual content and involved in final approval of the version to be submitted.

## FUNDING INFORMATION

This research did not receive any specific grant from funding agencies in the public, commercial or not‐for‐profit sectors.

## CONFLICT OF INTEREST STATEMENT

None.

## ETHICAL APPROVAL

This study was approved by the University Health Sciences Ethics Committee.

## PATIENT CONSENT

No patients were involved in this study.

## Data Availability

The data that support the findings of this study are available from the corresponding author upon reasonable request.

## References

[nop21810-bib-0001] Bengtsson, M. (2016). How to plan and perform a qualitative study using content analysis. NursingPlus Open, 2(1), 8–14.

[nop21810-bib-0002] Berndtsson, I. , Karlsson, M. G. , & Rejnö, Å. (2019). Nursing students' attitudes toward care of dying patients: A pre‐ and post‐palliative course study. Heliyon, 5(10), e02578. 10.1016/j.heliyon.2019.e02578 31667412 PMC6812234

[nop21810-bib-0003] Braun, V. , & Clarke, V. (2006). Using thematic analysis in psychology. Qualitative Research in Psychology, 3(2), 77–101. 10.1191/1478088706qp063oa

[nop21810-bib-0004] Braun, V. , & Clarke, V. (2021). Thematic analysis. SAGE Publications Ltd, UK.

[nop21810-bib-0005] Caswell, G. , Pollock, K. , Harwood, R. , & Porock, D. (2015). Communication between family carers and health professionals about end‐of‐life care for older people in the acute hospital setting: A qualitative study. BMC Palliative Care, 14, 35. 10.1186/s12904-015-0032-0 26231339 PMC4522056

[nop21810-bib-0006] Chua, J. , & Shorey, S. (2021). Effectiveness of end‐of‐life educational interventions at improving nurses and nursing students' attitude toward death and care of dying patients: A systematic review and meta‐analysis. Nurse Education Today, 101, 104892. 10.1016/j.nedt.2021.104892 33866077

[nop21810-bib-0008] Cooper, J. , & Barnett, M. (2005). Aspects of caring for dying patients which cause anxiety to first year student nurses. International Journal of Palliative Nursing, 11(8), 423–430. 10.12968/ijpn.2005.11.8.19611 16215518

[nop21810-bib-0009] DeJonckheere, M. , & Vaughn, L. M. (2019). Semistructured interviewing in primary care research: A balance of relationship and rigour. Family Medicine and Community Health, 7(2), e000057. 10.1136/fmch-2018-000057 32148704 PMC6910737

[nop21810-bib-0010] Dimoula, M. , Kotronoulas, G. , Katsaragakis, S. , Christou, M. , Sgourou, S. , & Patiraki, E. (2019). Undergraduate nursing students' knowledge about palliative care and attitudes towards end‐of‐life care: A three‐cohort, cross‐sectional survey. Nurse Education Today, 74, 7–14. 10.1016/j.nedt.2018.11.025 30554033

[nop21810-bib-0011] Ek, K. , Westin, L. , Prahl, C. , Osterlind, J. , Strang, S. , Bergh, I. , Henoch, I. , & Hammarlund, K. (2014). Death and caring for dying patients: Exploring first‐year nursing students' descriptive experiences. International Journal of Palliative Nursing, 20(10), 509–515. 10.12968/ijpn.2014.20.10.509 25350217

[nop21810-bib-0012] Faronbi, J. O. , Akinyoola, O. , Faronbi, G. O. , Bello, C. B. , Kuteyi, F. , & Olabisi, I. O. (2021). Nurses' attitude toward caring for dying patients in a Nigerian teaching hospital. SAGE Open Nursing., 7, 237796082110052. 10.1177/23779608211005213 PMC8047931 33912673

[nop21810-bib-0013] Garrino, L. , Contratto, C. , Massariello, P. , & Dimonte, V. (2017). Caring for dying patient and rheir families: The lived experiences of nursing students in Italy. Journal of Palliative Care, 32(3–4), 127–133. 10.1177/0825859717745169 29187085

[nop21810-bib-0014] Gillan, P. C. , Jeong, S. , & van der Riet, P. (2021). Embodied good deaths and disembodied bad deaths: Undergraduate nursing students' narratives of experience. Nurse Education Today, 97, 104674. 10.1016/j.nedt.2020.104674 33264738

[nop21810-bib-0015] Gillan, P. C. , van der Riet, P. J. , & Jeong, S. (2014). End of life care education, past and present: A review of the literature. Nurse Education Today, 34(3), 331–342. 10.1016/j.nedt.2013.06.009 23838297

[nop21810-bib-0016] Graneheim, U. H. , & Lundman, B. (2004). Qualitative content analysis in nursing research: Concepts, procedures and measures to achieve trustworthiness. Nurse Education Today, 24(2), 105–112. 10.1016/j.nedt.2003.10.001 14769454

[nop21810-bib-0017] Grubb, C. , & Arthur, A. (2016). Student nurses' experience of and attitudes towards care of the dying: A cross‐sectional study. Palliative Medicine, 30(1), 83–88. 10.1177/0269216315616762 26577928

[nop21810-bib-0018] Gwande, A. (2014). Being mortal. Aging, illness, medicine and what matters in the end. Palgrave Macmillan.

[nop21810-bib-0019] Hagelin, C. L. , Melin‐Johansson, C. , Ek, K. , Henoch, I. , Österlind, J. , & Browall, M. (2022). Teaching about death and dying—A national mixed‐methods survey of palliative care education provision in Swedish undergraduate nursing programmes. Scandinavian Journal of Caring Sciences, 36(2), 545–557. 10.1111/scs.13061 34962307

[nop21810-bib-0020] Hançer Tok, H. , & Cerit, B. (2021). The effect of creative drama education on first‐year undergraduate nursing student attitudes toward caring for dying patients. Nurse Education Today, 97, 104696. 10.1016/j.nedt.2020.104696 33388550

[nop21810-bib-0021] Henoch, I. , Melin‐Johansson, C. , Bergh, I. , Strang, S. , Ek, K. , Hammarlund, K. , Lundh Hagelin, C. , Westin, L. , Österlind, J. , & Browall, M. (2017). Undergraduate nursing students' attitudes and preparedness toward caring for dying persons – a longitudinal study. Nurse Education in Practice, 26, 12–20. 10.1016/j.nepr.2017.06.007 28648955

[nop21810-bib-0022] Heyland, D. , Cook, D. , Bagshaw, S. M. , Garland, A. , Stelfox, H. T. , Mehta, S. , Dodek, P. , Kutsogiannis, J. , Burns, K. , Muscedere, J. , Turgeon, A. F. , Fowler, R. , Jiang, X. , Day, A. G. , & Canadian Critical Care Trials Group.; Canadian Researchers at the End‐of‐Life Network . (2015). The very elderly admitted to ICU: A quality finish? Critical Care Medicine, 43(7), 1352–1360. 10.1097/CCM.0000000000001024 25901550

[nop21810-bib-0023] Hökkä, M. , Rajala, M. , Kaakinen, P. , Lehto, J. T. , & Pesonen, H. M. (2022). The effect of teaching methods in palliative care education for undergraduate nursing and medical students: A systematic review. International Journal of Palliative Nursing, 28(6), 245–253. 10.12968/ijpn.2022.28.6.245 35727833

[nop21810-bib-0024] Honkavuo, L. (2021). Ethics simulation in nursing education: Nursing students' experiences. Nursing Ethics, 969733021994188. Advance online publication., 1269–1281. 10.1177/0969733021994188 33827328

[nop21810-bib-0025] Hyde, A. , Lohan, M. , & McDonnell, O. (2004). Sociology for health professionals in Ireland. Institute of Public Administration.

[nop21810-bib-0026] Jablonski, A. , McGuigan, J. , & Westmoreland Miller, C. (2020). Innovative End‐of‐Life Simulation: Educating Nursing Students to Care for Patients During Transition. Innovative End‐of‐Life Simulation: Educating Nursing Students to Care for Patients During Transition.

[nop21810-bib-0027] Kallio, H. , Pietilä, A. M. , Johnson, M. , & Kangasniemi, M. (2016). Systematic methodological review: Developing a framework for a qualitative semi‐structured interview guide. Journal of Advanced Nursing, 72(12), 2954–2965. 10.1111/jan.13031 27221824

[nop21810-bib-0028] Kudubes, A. A. , Akıl, Z. K. , Bektas, M. , & Bektas, İ. (2021). Nurses' attitudes towards death and their effects on spirituality and spiritual care. Journal of Religion and Health, 60(1), 153–161. 10.1007/s10943-019-00927-2 31598824

[nop21810-bib-0029] Le, K. , Lee, J. , Desai, S. , Ho, A. , & van Heukelom, H. (2021). The surprise question and serious illness conversations: A pilot study. Nursing Ethics, 969733020983392. Advance online publication., 1010–1025. 10.1177/0969733020983392 33686904

[nop21810-bib-0030] Lippe, M. P. , & Becker, H. (2015). Improving attitudes and perceived competence in caring for dying patients: An end‐of‐life simulation. Nursing Education Perspectives, 36(6), 372–378. 10.5480/14-1540 26753296

[nop21810-bib-0031] McCall, K. (2018). Effects of transformational learning on student Nurses' perceptions and attitudes of caring for dying patients. Walden Dissertations and Doctoral Studies, 1–111. https://scholarworks.waldenu.edu/dissertations/5102

[nop21810-bib-0032] Muir, E. (1997). Ritual in early modern Europe. Cambridge University Press.

[nop21810-bib-0033] NICE . (2015). National Institute for health and care excellence. Care of dying adults in the last days of life https://www.nice.org.uk/guidance/ng31 26741019

[nop21810-bib-0034] NICE . (2019). National Institute for health and care excellence. End of Life Care for Adults: Service Delivery, 1–41. https://www.nice.org.uk/guidance/ng142 31633897

[nop21810-bib-0035] Nicol, J. , & Pocock, M. (2020). Memento Mori: Can art assist student nurses to explore death and dying? A Qualitative Study. Nurse Education Today, 89, 104404. 10.1016/j.nedt.2020.104404 32220697

[nop21810-bib-0036] Österlind, J. , Prahl, C. , Westin, L. , Strang, S. , Bergh, I. , Henoch, I. , Hammarlund, K. , & Ek, K. (2016). Nursing students' perceptions of caring for dying people, after one year in nursing school. Nurse Education Today, 41, 12–16. 10.1016/j.nedt.2016.03.016 27138476

[nop21810-bib-0037] O'Suilleabhain, S. (1997). Irish wake amusements. Mercier Press.

[nop21810-bib-0038] Parry, M. (2011). Student nurses' experience of their first death in clinical practice. International Journal of Palliative Nursing, 17(9), 446–451. 10.12968/ijpn.2011.17.9.448 22067736

[nop21810-bib-0039] Polit, D. F. , & Tatano Beck, C. (2014). Essentials of nursing research: Appraising evidence for nursing practice. Lippincott Williams & Wilkins.

[nop21810-bib-0040] Ranse, K. , Ranse, J. , & Pelkowitz, M. (2018). Third‐year nursing students' lived experience of caring for the dying: A hermeneutic phenomenological approach. Contemporary Nurse, 54(2), 160–170. 10.1080/10376178.2018.1461572 29669455

[nop21810-bib-0041] Ridge, A. (2009). Death customs in rural Ireland, traditional funerary rites in the Irish midlands. Arlen House.

[nop21810-bib-0042] Roxby, A. (2012). Are nursing rituals and superstitions helpful? Nursing Times Available at, 1. https://www.nursingtimes.net/students/are‐nursing‐rituals‐and‐superstitions‐helpful‐21‐05‐2012/

[nop21810-bib-0043] Sharour, L. A. , Suleiman, K. , Yehya, D. , Al‐Kaladeh, M. , Malak, M. , Subih, K. , & Bani, S. A. (2017). Nurses' students' attitudes toward death and caring for dying cancer patients during their placement. EuroMediterranean Biomedical Journal, 12(40), 189–193. 10.3269/1970-5492.2017.12.40

[nop21810-bib-0044] Shih, F. J. , Gau, M. L. , Lin, Y. S. , Pong, S. J. , & Lin, H. R. (2006). Death and help expected from nurses when dying. Nursing Ethics, 13(4), 360–375. 10.1191/0969733006ne881oa 16838568

[nop21810-bib-0045] Smith, M. B. , Macieira, T. G. , Bumbach, M. D. , Garbutt, S. J. , Citty, S. W. , Stephen, A. , Ansell, M. , Glover, T. L. , & Keenan, G. (2018). The use of simulation to teach nursing students and clinicians palliative care and end‐of‐life communication: A systematic review. American Journal of Hospice & Palliative Medicine, 35(8), 1140–1154. 10.1177/1049909118761386 29514480 PMC6039868

[nop21810-bib-0046] Smith‐Stoner, M. , Hall‐Lord, M. L. , Hedelin, B. , & Petzäll, K. (2011). Nursing students' concerns about end of life in California, Norway, and Sweden. International Journal of Palliative Nursing, 17(6), 271–277. 10.12968/ijpn.2011.17.6.271 21727884

[nop21810-bib-0047] Strang, S. , Bergh, I. , Ek, K. , Hammarlund, K. , Prahl, C. , Westin, L. , Österlind, J. , & Henoch, I. (2014). Swedish nursing students' reasoning about emotionally demanding issues in caring for dying patients. International Journal of Palliative Nursing, 20(4), 194–200. 10.12968/ijpn.2014.20.4.194 24763328

[nop21810-bib-0048] Taylor, L. J. (1989). Bas inEirinn: Cultural construction of death in Ireland. Anthropological Quarterly, 62(4), 175–187 ISSN 0003–5491.

[nop21810-bib-0049] Walter, T. , Littlewood, J. , & Pickering, M. (1995). Death in the news: The public invigilation of private emotion. Sociology, 29(4), 579–596. 10.1177/0038038595029004002

[nop21810-bib-0050] Wang, Y. (2019). Nursing students' experiences of caring for dying patients and their families: A systematic review and meta‐synthesis. Frontiers of Nursing, 6(4), 261–272. 10.2478/FON-2019-0042

[nop21810-bib-0051] Westwood, S. , & Brown, M. (2019). Preparing students to care for patients at the end of life. Nursing Times [Online], 115: 10, 43–46. 10.1016/j.npls.2016.01.001

[nop21810-bib-0052] WHO . (2020). World health organisation. Palliative Care https://www.who.int/news‐room/fact‐sheets/detail/palliative‐care

[nop21810-bib-1047] Yoong, S. Q. , Wang, W. , Seah, A. C. W. , Kumar, N. , Gan, J. O. N. , Schmidt, L. T. , Lin, Y. , & Zhang, H. (2023). Nursing students' experiences with patient death and palliative and end‐of‐life care: A systematic review and meta‐synthesis. Nurse Education in Practice, 69, 103625. 10.1016/j.nepr.2023.103625 37004470

[nop21810-bib-0053] Younas, A. (2020). Relational inquiry approach for developing deeper awareness of patient suffering. Nursing Ethics, 27(4), 935–945. 10.1177/0969733020912523 32249677

[nop21810-bib-0054] Zheng, R. , Lee, S. F. , & Bloomer, M. J. (2018). How nurses cope with patient death: A systematic review and qualitative meta‐synthesis. Journal of Clinical Nursing, 27(1–2), e39–e49. 10.1111/jocn.13975 28748639

[nop21810-bib-0055] Zhou, Y. , & Wei Zhnag, Q. L. (2020). Undergraduate nursing students' knowledge, attitudes and self‐efficacy regarding palliative care in China: A descriptive correlational study. Nursing Open, 8(1), 343–353. 10.1002/nop2.635 33318842 PMC7729553

[nop21810-bib-0056] Zulfatual, M. , Setioputro, B. , & Kurniawan, E. (2018). Nursing student's attitudes towards caring for dying patients. Nurse Media Journal of Nursing, 8(1), 25–34. 10.14710/nmjn.v8i1.17270

